# Exploratory analysis of the neutrophil to lymphocyte ratio in patients with pulmonary arterial hypertension

**DOI:** 10.1186/s12890-017-0407-5

**Published:** 2017-04-26

**Authors:** Lars Harbaum, Kaaja M. Baaske, Marcel Simon, Tim Oqueka, Christoph Sinning, Antonia Glatzel, Nicole Lüneburg, Karsten Sydow, Carsten Bokemeyer, Hans Klose

**Affiliations:** 1 Center of Pulmonary Arterial Hypertension Hamburg, University Medical Center Hamburg-Eppendorf, Hamburg, Germany; 2 Section Pneumology, Department of Medicine II, University Medical Center Hamburg-Eppendorf, Hamburg, Germany; 3 Department of General and Interventional Cardiology, University Heart Center Hamburg, Hamburg, Germany; 4 Institute of Clinical Pharmacology and Toxicology, University Medical Center Hamburg-Eppendorf, Hamburg, Germany; 5 Oncology, Hematology and Stem Cell Transplantation, Department of Medicine II, University Medical Center Hamburg-Eppendorf, Hamburg, Germany

**Keywords:** Pulmonary hypertension, Pulmonary arterial hypertension, Inflammation, White blood cell count, Neutrophils, Granulocytes, Eosinophils, C-reactive protein, Survival, Prognosis

## Abstract

**Background:**

Chronic inflammation emerges as a feature of the pathogenesis of pulmonary arterial hypertension (PAH) in experimental models. Alterations of circulating cell subsets have been observed in patients with PAH. We aimed to assess associations of the white blood cell count with disease severity and outcome in patients with PAH.

**Methods:**

The total and differential white blood cell count was related to functional parameters, pulmonary hemodynamics and transplantation-free survival in 77 patients with PAH in an observational single center study.

**Results:**

An increased neutrophil/lymphocyte ratio was associated with poor World Health Organization functional class and shorter 6-minute walking distance, as well as with elevated right atrial pressure and high level of N-terminal prohormone of brain natriuretic peptide. During a median follow-up period of 31 months (range 16-56) 23 patients died and 2 patients were referred to lung transplantation. Using uni- and subsequent bivariate Cox proportional hazards analyses an increased neutrophil/lymphocyte ratio was associated with unfavorable transplantation-free survival independent of hemodynamic parameters and C-reactive protein. The prognostic implication sustained in subsets of patients with incident PAH and in the absence of cardiovascular risk factors.

**Conclusions:**

The results of this analysis indicate that a neutrophilic inflammation may be associated with clinical deterioration and poor outcome in patients with PAH. Assessing the composition of the differential white blood cell count may render prognostic information and could represent a step towards incorporating an inflammatory marker into the clinical management of patients with PAH.

**Electronic supplementary material:**

The online version of this article (doi:10.1186/s12890-017-0407-5) contains supplementary material, which is available to authorized users.

## Background

Pulmonary arterial hypertension (PAH) is a rare, progressive vascular disease characterized by elevated pulmonary vascular resistance and right heart failure [1]. The pathomechanism of PAH consists of endothelial dysfunction, in-situ thrombosis, occlusive vascular remodeling with loss of small vessels and perivascular inflammation including the development of tertiary lymphoid follicles [2, 3]. The importance of the immune system to regulate pulmonary vascular homeostasis has been demonstrated. In experimental models of pulmonary hypertension (PH), vascular inflammation may even precede vascular remodeling [3–5]. Accumulation of circulating cytokines such as interleukin (IL) 2, 6, 8, 10 and 12p70 and C-reactive protein (CRP) has been associated with poor overall survival in patients with PAH [6–8]. However, the inflammatory state found in patients with PAH has not yet been incorporated into the clinical management of the disease.

Parameters of the differential white blood cell (WBC) count have been linked to poor outcome in cardiovascular diseases. In patients with coronary heart disease or chronic left heart failure, increased neutrophils, decreased lymphocytes and particularly an increased neutrophil/lymphocyte ratio have been related to poor outcome [9–11]. The ratio of the absolute numbers of neutrophils and lymphocytes is a simple and readily assessable measure of the inflammatory state, which may be considered more stable than the individual cell counts and less affected by acute conditions [11]. To date, there is only one study assessing the clinical significance of the neutrophil/lymphocyte ratio in patients with PAH. The study demonstrated that an increased neutrophil/lymphocyte ratio was associated with poor functional class and elevated mortality. The prediction of outcome, however, was not independent of other parameters of disease severity [12].

The present study was conducted to assess the association of total and differential WBC count parameters with functional and hemodynamic parameters as well as transplantation-free survival in patients with PAH.

## Methods

### Study design

A retrospective observational study was conducted at the Center of Pulmonary Arterial Hypertension Hamburg at the University Medical Center Hamburg-Eppendorf, Germany, identifying patients with (1) age ≥ 18 years, (2) diagnosis of PAH according to current guidelines [1] and (3) admission to our hospital for right heart catheterization between 1 January 2010 and 31 December 2013. To perform right heart catheterization patients are routinely admitted to our hospital for at least one day. In case more than one right heart catheterization was performed during the study period, data of the first examination was retrieved for analysis. Patients with current infection and anti-infective treatment were excluded. Infection was assessed by means of clinical signs and symptoms, which have to lead to an anti-infective treatment (e.g., antibiotic or antiviral therapy). The Ethics Committee of the Hamburg Chamber of Physicians waived the need to obtain consent for the collection, analysis, and publication of the retrospectively obtained and anonymized data for this non-interventional study.

### Data collection

Data were collected from the electronic patient data management system (Soarian Clinicals 4.00, Cerner Health Services, North Kansas City, MO, USA). Hemodynamic parameters included right atrial pressure (RAP), mean pulmonary arterial pressure (mPAP), pulmonary arterial wedge pressure (PAWP) and oxygen saturation of mixed venous blood (SvO_2_). Cardiac output (CO) was determined by the direct Fick method. Pulmonary vascular resistance (PVR) and cardiac index (CI) were calculated using standard formulas. Parameters of disease severity and laboratory testing were usually obtained within one week before or after right heart catheterization. Parameters of disease severity included 6-minute walking distance (6MWD), WHO functional class (WHO-FC) and N-terminal of the prohormone brain natriuretic peptide (NT-proBNP). Differential WBC count was obtained by standard laboratory procedure (Advia 2120i, Siemens, Munich, Germany) and included the analysis of leukocytes, lymphocytes, granulocytes (neutrophils, basophils and eosinophils) and monocytes. The detection threshold for eosinophils and basophils was 0.1 10^6^ cells/ml. The neutrophil/lymphocyte ratio was calculated using absolute numbers of cells. Furthermore, erythrocytes, thrombocytes, CRP and serum creatinine were obtained. The detection threshold for CRP was 5 mg/dl. Glomerular filtration rate (eGFR) was estimated using the modification of diet in renal disease equation (ml/min/1.73 m^2^). Co-morbidities including systemic arterial hypertension, diabetes, coronary heart disease, atrial fibrillation and chronic obstructive pulmonary disease were also retrieved from the database. The sum of these co-morbidities were obtained as described previously in the AMBITION trial [13].

Treatment with targeted PAH-medication was commenced as approved and recommended during the observation period [14, 15]. In our PAH referral center a goal-oriented treatment strategy has been applied using endothelin receptor antagonists (ERA), phosphodiesterase-5 (PDE-5) inhibitors and prostacyclin derivatives in a varying order. The combined outcome endpoint was death or lung transplantation. Transplantation-free survival was recorded by reviewing patients’ charts and/or by contacting the attending general physician.

### Data analysis

Differences of means between unpaired samples were assessed by unpaired Student’s *t*-test for parametric and Mann–Whitney-*U* test for non-parametric data. Chi-square test was used to compare ordinal data. For bivariate correlations Pearson correlation was performed to assess parametric data and Spearman’s rank correlation for non-parametric data.

Receiver operating characteristic (ROC) analyses were performed across the ranges of differential WBC count parameters (lymphocytes, neutrophils and neutrophil/lymphocyte ratio). Values with the highest sum of sensitivity and specificity were obtained as cut-offs for subsequent survival analyses. In addition, in survival analyses values of 6MWD > 380 m was selected as cut-offs due to their prognostic significance at baseline [1, 16]. As thresholds for NT-proBNP at baseline the mean value was used. As threshold for GFR stage 3 to 5 or renal impairment according to the National Kidney Foundation (≤ 60 ml/ml/1.73 m^2^) were applied [17]. Survival was assessed by Kaplan-Meier analyses and compared by log-rank test. For univariable and bivariable testing a Cox proportional hazard regression model was used. All *p*-values were two-sided and a *p*-value of less than 0.05 was considered significant. All statistical analyses were performed with SPSS statistics 20 software (IBM, Armonk, New York, United States).

## Results

### Characteristics of patients

Overall, 104 patients with PAH were identified. Six patients were excluded due to current infectious disease (2 pneumonia, 2 urinary tract infection and 2 HIV-infections with antiretroviral therapy). In a next step 21 patients were excluded due to unavailability of differential WBC, leading to a final study cohort of 77 patients. In this cohort 54 patients (70%) were diagnosed with idiopathic PAH (IPAH), 2 patients (3%) with hereditary PAH (HPAH), 1 patient (1%) with drug induced PAH and 20 patients (26%) with associated PAH (APAH). APAH was due to connective tissue disease in 12 patients (60%), due to porto-pulmonary hypertension in 2 patients (10%) and due to congenital heart disease in 3 patients (15%). In 1 patient the diagnosis of IPAH at time of right heart catheterization was later revised to HIV-associated PAH. The diagnosis of PAH was prevalent in 41 patients (53%), of which, on the day of right heart catheterization, 20 patients (49%) were treated with an ERA, 30 patients (73%) with a PDE-5 inhibitor and 13 patients (32%) with a prostacyclin derivative. The half of patients (51%) was on combination therapy. The majority of patients (65%) were in WHO-FC III and a WHO-FC I or II was present in only 15 patients (20%). Demographic, functional and hemodynamic parameters are shown in Table 1. Differential blood count values are summarized in Table 2. Eosinophils above the threshold of 0.1 10^6^ cells/l were detected in 40 patients (41%), while basophils above the threshold of 0.1 10^6^ cells/ml were elevated in 3 patients (3%) only.

**Table 1:** Demographic, functional and hemodynamic characteristics of patients with pulmonary arterial hypertension (PAH)

Parameters	PAH patients (*n* = 77)
Age, years	61 ± 14
Gender female/male	58/19
WHO-FC I/II/III/IV	2/13/50/12
6MWD, m	355 ± 135
NT-proBNP, ng/l	3274 ± 4750
GFR, ml/min/1.73 m^2^	72 ± 50
CRP > 5/≤ 5, mg/dl	26/50
mPAP, mm Hg	43 ± 16
RAP, mm Hg	9 ± 5
PAWP, mm Hg	12 ± 6
PVR, dyn · s · cm^−5^	822 ± 645
SvO_2_, %	62 ± 12
CI, l/min/m^2^	2.21 ± 1.14
RVSP, mm Hg	59 ± 23
TAPSE, mm	18 ± 6
Arterial hypertension	32 (42%)
Diabetes mellitus	10 (13%)
Coronary heart disease	12 (16%)
Atrial fibrillation	15 (20%)
BMI ≥ 30 kg/m^2^	17 (22%)
CVRF ≥ 3^a^	7 (9%)
Chronic obstructive pulmonary disease	16 (21%)

Data are presented as mean ± standard deviation or numbers. ^a^CVRF including arterial hypertension, diabetes mellitus, coronary heart disease, atrial fibrillation and body mass index

*6MWD* 6 minute walking distance, *CVRF *cardiovascular risk factors, *CI* cardiac index, *CRP* C-reactive protein, *GFR* glomerular filtration rate, *mPAP* mean pulmonary arterial pressure, *NT-proBNP* N-terminal of the prohormone brain natriuretic peptide, *PVR* pulmonary vascular resistance, *RAP* right atrial pressure, *RVSP* right ventricular systolic pressure, *SvO*_*2*_ oxygen saturation of mixed venous blood, *TAPSE* tricuspid annular plane systolic excursion, *WHO-FC* World Health Organization functional class

**Table 2:** Differential blood count parameters of patients with pulmonary arterial hypertension (PAH)

Parameters	PAH patients (*n* = 77)
Hemoglobin, mg/dl	13.38 ± 2.02
Erythrocytes, 10^6^/ml	4.46 ± 0.76
Leukocytes, 10^6^/ml	7.89 ± 3.12
Lymphocytes, 10^6^/ml	1.55 ± 0.71
Lymphocytes, %	21.26 ± 9.18
Neutrophils, 10^6^/ml	5.46 ± 3.05
Neutrophils, %	68.24 ± 11.93
Neutrophil/lymphocyte ratio	4.94 ± 7.43
Monocytes, 10^6^/ml	0.55 ± 0.43
Monocytes, %	6.88 ± 2.41
Eosinophils, 10^6^/ml	0.24 ± 0.14^a^
Eosinophils, %	5.05 ± 3.22^a^
Eosinophils > 0.1 · 10^6^/ml	40 (52%)
Basophils, 10^6^/ml	0.14 ± 0.02^b^
Basophils, %	1.63 ± 0.99^b^
Basophils > 0.1 · 10^6^/ml	3 (4%)
Thrombocytes, 10^6^/ml	235.45 ± 116.83

Data are presented as mean ± standard deviation or numbers (percentage)

^a^Patients with eosinophils > 0.1 10^6^/ml (*n* = 40)

^b^Patients with basophils > 0.1 · 10^6^/ml (*n* = 3)

### Correlation with functional and hemodynamic parameters

**Fig. 1:**
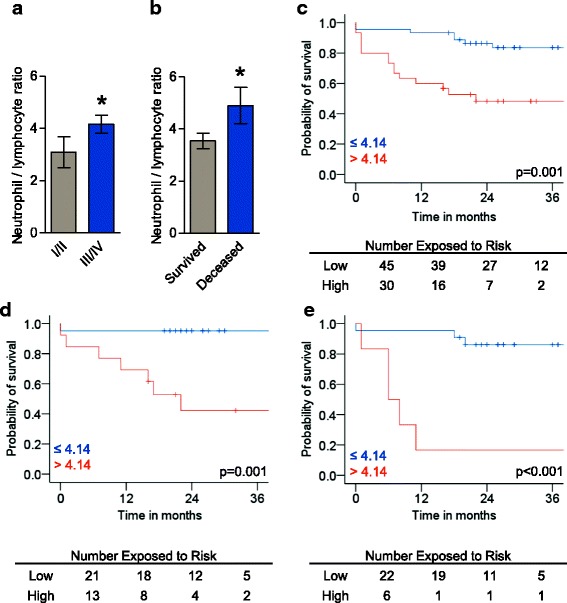
Association of the neutrophil/lymphocyte ratio with functional class and transplantation-free survival in patients with pulmonary arterial hypertension (PAH): Patients with WHO-FC of III/IV compared to I/II showed a higher neutrophil/lymphocyte ratio (*p* = 0.0281, **a**). Similarly the neutrophil/lymphocyte ratio was higher in patients who deceased or were referred to lung transplantation (*p* = 0.0394, **b**). Kaplan-Meier curves revealed an unfavorable transplantation-free survival for patients with a neutrophil/lymphocyte ratio > 4.14 (*p* = 0.001, **c**). This sustained in subsets of patients with incident disease (*p* = 0.001, **d**) or in the absence of any cardiovascular risk factors (CVRF,*p* < 0.001, **e**) applying the same cut-off. CVRF including arterial hypertension, diabetes mellitus, coronary heart disease, atrial fibrillation and body mass index greater of equal 30 kg/m^2^. CVRF = cardiovascular risk factors. WHO-FC = World Health Organization functional class

An exploratory correlation analysis of the WBC count with functional and hemodynamic parameters displayed a possible link between increased relative numbers of neutrophils and the neutrophil/lymphocyte ratio with disease severity. These parameters showed the highest correlation coefficients ranging between 0.348 and 0.443, and correlated inversely with the 6MWD and RAP as well as positively with the NT-proBNP level (Additional file 1: Table S1). In addition, the neutrophil/lymphocyte ratio was significantly related to WHO-FC. Patients with WHO-FC III/IV showed higher level of the neutrophil/lymphocyte compared to WHO-FC I/II (*p* = 0.0281, Fig. 1a).

Basophils and eosinophils were detectable only in small subsets of patients and were not included in correlation analyses. No association of basophils with clinical parameters occurred. However, patients with elevated numbers of eosinophils (above the threshold of 0.1 10^6^ cells/ml) showed a better 6MWD and less severe hemodynamic impairment as indicated by lower mPAP, PVR and higher SvO_2_. Elevated levels of eosinophils were not related with increased numbers of leukocytes, neutrophils nor lymphocytes (Additional file 2: Table S2).

### Association with transplantation-free survival

Follow-up was available for 76 out of 77 patients. During a median follow-up period of 31 months (range 16-56) 23 patients died and 2 patients were referred to lung transplantation. The 1-, 2- and 3-year overall transplantation-free survival rates were 80, 71 and 69%, respectively. In univariable analyses the relative numbers of neutrophils and the neutrophil/lymphocyte ratio revealed the strongest association of the WBC parameters and the combined outcome of either death or lung transplantation (Table 3). In the step, we peformed bivariate analyses with these WBC parameters and co-variables. Co-variables with *p* values less than 0.01 in univariable analyses were selected for subsequent bivariate models, which compared these co- variables with either the relative number of neutrophils or the neutrophil/lymphocyte ratio (GFR and WHO-FC,Table 4). CRP was selected as co-variable due to its potential to incorporate a routine inflammatory marker in the clinical assessment of patients with PAH. To account for possible confounding cardiovascular risk factors we selected the sum of risk factors including arterial hypertension, diabetes mellitus, coronary heart disease, atrial fibrillation and body mass index as co-variable for bivariate analysis. Furthermore to account for the heterogeneity of the cohort the subtype of PAH was also selected as co-variable for bivariate analyses as either idiopathic or non-idiopathic. The prognostic implication of the relative number of neutrophils was found to be independent of the selected variables including WHO-FC, CRP, GFR, SvO_2_ the sum of risk factors and the subtype of PAH in this cohort of patients. Moreover, the prognostic implication of the neutrophil/lymphocyte ratio was independent of the CRP, GFR, SvO_2,_ the sum of risk factors and the subtype of PAH in bivariate analyses (Table 4).

**Table 3:** Univariable Cox’s proportional hazards regression analyses regarding transplantation-free survival in patients with pulmonary arterial hypertension (PAH)

Variable	Univariable Cox’s proportional hazards regression model		
	HR	95% CI	*p*-value
Neutrophils, 10^6^/ml	1.03	0.92–1.14	0.644
Neutrophils, %	1.06	1.02–1.1	0.002
Lymphocytes, 10^6^/ml	0.37	0.17–0.83	0.016
Lymphocytes, %	0.22	0.90–0.99	0.022
Neutrophil/lymphocyte ratio	1.05	1.01–1.08	0.006
Eosinophils, > 0.1 10^6^/ml	0.79	0.35–1.77	0.537
Age, years	1.01	0.98–1.04	0.454
Male gender	1.42	0.68–2.96	0.346
Prevalent vs. incident PAH	0.70	0.36–1.36	0.296
Idiopathic PAH vs. non-idiopathic PAH	2.17	1.11–4.24	0.023
WHO-FC	2.62	1.32–5.15	0.005
6WMD, >380 m	0.29	0.10–0.87	0.027
NT-proBNP, > 3474 ng/l	2.33	1.19–4.57	0.014
CRP > 5 mg/dl	2.05	1.04–4.05	0.039
GFR > 60 ml/min/1.73 m^2^	0.38	0.19–0.74	0.004
mPAP, mm Hg	1.02	0.99–1.04	0.142
Cardiac index, ml/min/m^2^	0.58	0.55–1.39	0.580
PVR, dyn*s*cm^−5^	1	1–1.01	0.13
RAP, mm Hg	1.06	0.99–1.14	0.108
SvO_2_, %	0.96	0.96–0.99	0.007
Arterial hypertension	0.78	0.39–1.54	0.470
Diabetes mellitus	0.76	0.27–2.17	0.618
Coronary heart disease	1.67	0.80–3.46	0.171
Atrial fibrillation	0.91	0.34–2.46	0.858
BMI ≥ 30 kg/m^2^	1.05	0.39–2.81	0.92
CVRF ≥ 3^a^	0.76	0.18–3.27	0.716
Chronic obstructive pulmonary disease	0.67	0.26–1.72	0.405

^a^CVRF including arterial hypertension, diabetes mellitus, coronary heart disease, atrial fibrillation and body mass index

*6MWD* 6 minute walking distance, *BMI* body mass index, *CI* confidence interval, *CRP* C-reactive protein, *CVRF* cardiovascular risk factors, *GFR* glomerular filtration rate, *HR* hazard ratio, *mPAP* mean pulmonary arterial pressure, *NT-proBNP* N-terminal of the prohormone brain natriuretic peptide, *PVR* pulmonary vascular resistance, *RAP* right atrial pressure, *SvO*_*2*_ mixed venous oxygen saturation, *WHO-FC* World Health Organization functional class

**Table 4:** Bivariable Cox’s proportional hazards regression analyses regarding transplantation-free survival

Variable	Bivaribale Cox’s proportional hazards regression model^a^					
	HR, CI and *p*-value of variable with neutrophils in %			HR, CI and *p*-value of neutrophils in % with variable		
	HR	95% CI	*p*-value	HR	95% CI	*p*-value
CRP > 5 mg/dl	1.97	0.86–4.54	0.112	1.05	1.01–1.08	0.014
CVRF ≥ 3^b^	0.56	0.13–2.43	0.443	1.06	1.02–1.09	0.002
WHO-FC	2.13	1.06–4.23	0.034	1.04	1.01–1.08	0.021
GFR > 60 ml/min/1.73 m^2^	1.8	0.75–4.31	0.188	1.04	1.01–1.08	0.019
SvO_2_, %	0.97	0.95–0.99	0.013	1.05	1.01–1.1	0.015
Idiopathic vs. non-idiopathic	1.47	0.62–3.48	0.382	1.06	1.02–1.09	0.002
	HR, CI and *p*-value of variable with neutrophils/lymphocytes ratio			HR, CI and *p*-value of neutrophils/lymphocytes ratio with variable		
	HR	95% CI	*p*-value	HR	95% CI	*p*-value
CRP > 5 mg/dl	2.56	1.13–5.82	0.025	1.05	1.02–1.09	0.004
CVRF ≥ 3^b^	0.31	0.04–2.47	0.265	1.06	1.02–1.11	0.007
WHO-FC	2.23	1.17–4.67	0.016	1.03	1–1.07	0.064
GFR > 60 ml/min/1.73 m^2^	2.17	0.95–4.99	0.067	1.04	1–1.07	0.033
SvO_2_, %	0.97	0.95–0.99	0.013	1.04	1.01–1.08	0.015
Idiopathic vs. non-idiopathic	1.19	0.49–2.9	0.706	1.04	1.01–1.08	0.012

^a^Separate bivariate models for each given parameter with either relative count of neutrophils or the neutrophil/lymphocyte ratio

^b^CVRF including arterial hypertension, diabetes mellitus, coronary heart disease, atrial fibrillation and body mass index

*CI* confidence interval, *CRP* C-reactive protein, *CVRF* cardiovascular risk factors, *GFR* glomerular filtration rate, *HR* hazard ratio, *SvO2* oxygen saturation of mixed venous blood, *WHO-FC* World Health Organization functional class

Receiver operating characteristics (ROC) revealed a discriminating potential of the neutrophil/lymphocyte ratio between transplantation-free survival status with superior area under the curve as compared to the relative number of neutrophils (Additional file 3: Figure S1A). The neutrophil/lymphocyte ratio was higher in patients who deceased or were referred to lung transplantation (*p* = 0.0394, Fig. 1b). The threshold of the neutrophil/lymphocyte ratio with the highest sum of sensitivity and specificity derived from ROC analysis was 4.14 in the overall group of patients (sensitivity 40%, specificity 71%), which significantly stratified survival in a Kaplan-Meier curve (Fig. 1c). This cut-off was also associated with functional and hemodynamic impairment of patients (Additional file 4: Table S3). Taking out subsets of patients, the neutrophil/lymphocyte ratio proved to be significantly associated with survival status in incident patients (*n* = 36) and in patients without any cardiovascular risk factor (*n* = 28, Additional file 3: Figure S1B-C). Cut-off values derived from these ROC analyses were equal to the overall group (both 4.14) and significantly stratified survival (Fig. 1d-e). In patients with idiopathic disease (*n* = 54) the neutrophil/lymphocyte ratio was not associated with survival status in this cohort of patients (Additional file 3: Figure S1D-E).

## Discussion

The present study revealed that increased relative number of neutrophils and particularly increased neutrophil/lymphocyte ratio were associated with more severe disease and poor outcome in patients with PAH. On the other hand, the absolute number of cell count was not found to be associated with disease severity and survival. The prediction of transplantation-free survival of the neutrophil/lymphocyte ratio was found to be independent of functional, hemodynamic parameters, GFR, CRP levels and co-morbidities in the present cohort of patients. This implication on the outcome sustained significant in subsets of patients with incident PAH or in PAH patients without co-morbidities known for their risk for left heart disease, but not in patients with only idiopathic disease. In addition to these findings, we observed that elevated eosinophils were associated with impaired hemodynamics and functional capacity, but not with outcome. Elevated eosinophils were found in patients with lower mPAP and PVR. Although this is, to our knowledge, the first clinical analysis of circulating eosinophils in patients with PAH, these findings challenge the observations derived from experimental PH and need to be validated.

Clinical management of patients with PAH involves the assessment of circulating biomarkers that reveal severity of pulmonary vascular disease by reflecting the impairment of related organs such as right heart or renal failure [1, 16]. Levels of NT-proBNP for instance are elevated following right ventricular impairment and reflect the severity of pulmonary vascular flow obstruction in patients with PAH [18]. Impaired renal function may represent decreased systemic perfusion as a consequence of decreased cardiac output [18]. Among hematological parameters, an increased red blood cell distribution width (RDW) was independently associated with increased mortality in patients with PAH [19, 20]. In a cohort of patients with idiopathic PAH, RDW was even superior to NT-proBNP and 6WMD in predicting mortality [19]. The RDW may be linked to ineffective erythropoiesis, hemolysis, chronic inflammation and/or iron-deficiency. Although, it is now increasingly recognized that the immune system is involved in the pathogenesis of patients with PAH, marker of the inflammatory state has not yet been incorporated into clinical management [1, 3].

Severe disease and higher mortality in PAH patients have been associated with increased circulating CRP levels in PAH [8]. Despite that the neutrophil/lymphocyte ratio correlated with CRP level, which has also been shown previously [21], its prediction of outcome was independent of the level of CRP in our cohort of patients. Thus, the total and differential WBC count, which represents an inexpensive and readily assessable parameter of inflammation, may render additional information on the disease state and patients’ outcome. In cardiovascular diseases, a ratio shifted towards an increased neutrophil/lymphocyte ratio has been observed to be associated with more severe diseases such as acute coronary syndrome or ischemic heart disease [9–11]. When added to the Framingham risk score in a general population cohort, the neutrophil/lymphocyte ratio reclassified those with a intermediate risk category as having lower or higher probability of cardiovascular mortality [11]. Explanation has here been suggested by the pro-atherogenic and pro-inflammatory effect of neutrophils on one side and the regulatory, quiescent action of lymphocytes (i.e., subsets) on the other side [10, 11, 22]. In addition, neutrophil/lymphocyte ratio seems to be increased among diabetic patients and in these it was also shown to be independently associated with cardiovascular events [23, 24]. The co-occurrence of cardiovascular risk factors has been increasingly observed alongside with a demographical shift towards older age in patients diagnosed with PAH who had entered clinical trials and registries [25]. This has led to the use of terms such as ‘atypical PAH’, which refers to patients who have 3 or more risk factors for left heart disease [25]. In a subsequent subset of patients with no CVRF (including arterial hypertension, diabetes mellitus, coronary heart disease, atrial fibrillation and body mass index ≥ 30 kg/m^2^) the neutrophil/lymphocyte ratio remained associated with survival in our cohort of PAH patients. Comparable to the results from the international Comparative, Prospective Registry of Newly Initiated Therapies for Pulmonary Hypertension (COMPERA) registry [25] the outcome of PAH with 3 or more CVRF in our cohort was similar to those patients with less than 3 CVRF.

Several studies have reported on metabolic remodeling as a feature in the diseased pulmonary vascular in PAH. Central to this is the energy metabolism in mitochondria, which is also implicated in other metabolic diseases such as diabetes. For instance, suppression of mitochondrial function with a shift towards glycolysis by loss of the mitochondrial deacetylase sirtuin 3 or by loss of the proton transporter uncoupling protein 2 (UCP2) led to spontaneous development of PH in mice [26, 27]. Interestingly, insulin resistance is also present in experimental PH of BMPR2 mutated mice, the predominant cause of hereditary PAH in humans [28].

In the pulmonary arteries neutrophils may release cytokines, enzymes and other factors when passing the pulmonary circulation. For instance, neutrophil elastase, an enzyme also produced by pulmonary vascular smooth muscle cells (SMC), can liberate growth factors from the extracellular matrix [29]. Inhibition of elastase has been shown to prevent and also reverse experimental PH by inducing apoptosis of SMC [30]. Interestingly, it has been demonstrated that neutrophils isolated from patients with IPAH showed an increased release of mediators such as elastase or leukotriene B4 compared with neutrophils from healthy control subjects [31]. Leukotriene B4 levels were also found to elevated in patients with PAH and its inhibition was shown to reverse experimental PH [32].

An altered composition of lymphocytes, particularly within the T helper cell compartment has been found in patients with PAH [3]. Deficiency of T cells in an athymic rat led to a particular aggressive type of experimental PH, probably due to the lack of regulatory T cells which are sufficient to self-limit vascular inflammation [4]. On the other hand, activation of the nuclear factor of activated T-cells (NFAT), which in PAH occurs though expression of Moloney murine leukemia virus (Pim-1) not only in T cells but also in pulmonary vascular SMC, was reported to induce a proproliferative and antiapoptotic phenotype of pulmonary vascular cells [33]. Interestingly, circulating level of Pim-1 has been found to be associated with disease severity and outcome in patients with PAH [34]. Here we observed that the number of circulating lymphocytes as retrieved from the WBC count is a less strong prognostic factors than the number of circulating neutrophils or the neutrophil/lymphocyte ratio.

Activation of the Th2 pathway by immunization and prolonged intermittent challenge via the airways induced severe muscularization of small- to medium-sized pulmonary arteries [35]. In addition, it has been shown that the presence of eosinophils is necessary for vascular remodeling in experimental PH induced by allergic inflammation [36]. However, the role of eosinophils and the Th2 pathway in non-allergic experimental PH are not clear. For instance, the presence of eosinophils in cancer is associated with less aggressive tumor biology and better outcome indicating an anti-mitogenic effect [37].

Foremost, limitations are those inherent to the retrospective observational study design. Further studies are now needed to confirm present results and to evaluate the underlying pathophysiological mechanisms. Particularly in the context of an underlying disease associated with PAH such as connective tissue disease, since our results base on a mixed cohort of patients with different form of PAH. Moreover, the WBC count parameters represent a snapshot of the inflammatory state. Longitudinal data of the WBC count were not available for this analysis. There are no established cut-off values for the WBC parameters. However, the cut-off applied in our analyses was consistent with prior findings. For instance studies showed that a neutrophil/lymphocyte ratio > 4.7 and > 4.5 predicted mortality in left heart disease [11, 38]. Unlike previous studies we did not observe a prognostic implication of PVR, CI or RAP in our cohort of patients [16].

## Conclusion

Our results indicate that neutrophilic inflammation with high relative number of neutrophils and a high neutrophil/lymphocyte ratio may be associated with functional and hemodynamic deterioration, as well as with elevated mortality or the need for lung transplantation. The observed prediction of outcome may be independent of functional and hemodynamic parameters, co-morbidities and the level of CRP. These findings may further incorporate the inflammatory state found in patients with PAH into the clinical perspective of the disease. The WBC count represents an inexpensive and readily assessable parameter of inflammation, which may be able to add relevant prognostic information to the clinical management of patients with PAH. However, to be considered as parameter in the routine assessment of patients with PAH further studies and validation are required.

## Additional files

Additional file 1: Table S1. Correlation of differential blood count parameters with functional and hemodynamic parameters of patients with PAH. (DOCX 13 kb)

Additional file 2: Table S2. Association of eosinophils with demographic, functional and hemodynamic as well as with differential blood count parameters in patients with PAH. (DOCX 13 kb)

Additional file 3: Figure S1. Receiver operating characteristics (ROC) across transplantation-free survival: ROC analyses across the range of relative numbers of neutrophils and the neutrophil/lymphocyte ratio in all patients with PAH, in patients with incident PAH and in patients with PAH without cardiovascular risk factors. (PDF 285 kb)

Additional file 4: Table S3. Association of the neutrophil/lymphocyte ratio with demographic, functional and hemodynamic parameters, as well as co-morbidities. (DOCX 16 kb)

## Abbreviations

6MWD: 6-minute walking distance
APAH: Associated pulmonary arterial hypertension
BMI: Body mass index
BMPR2: Bone morphogenetic protein receptor type 2
CI: Cardiac index
CO: Cardiac output
COMPERA: Comparative, prospective registry of newly initiated therapies for pulmonary hypertension
CRP: C-reactive protein
CVRF: Cardiovascular risk factors
eGFR: Estimated glomerular filtration rate
ERA: Endothelin receptor antagonists
GFR: Glomerular filtration rate
HbA1c: Hemoglobin A1c
HIV: Human immunodeficiency virus
HPAH: Hereditary pulmonary arterial hypertension
IL: Interleukin
IPAH: Idiopathic pulmonary arterial hypertension
mPAP: Mean pulmonary arterial pressure
NFAT: Nuclear factor of activated T-cells
NT-proBNP: N-terminal of the prohormone brain natriuretic peptide
PAH: Pulmonary arterial hypertension
PAWP: Pulmonary arterial wedge pressure
PDE-5: Phosphodiesterase-5
PH: Pulmonary hypertension
Pim-1: Provirus integration site of Moloney murine leukemia virus
PVR: Pulmonary vascular resistance
RAP: Right atrial pressure
RDW: Red blood cell distribution width
ROC: Receiver operating characteristic
RVSP: Right ventricular systolic pressure
SMC: Smooth muscle cells
SvO2: Oxygen saturation of mixed venous blood
TAPSE: Tricuspid annular plane systolic excursion
Th2: T-helper 2 cell
UPC2: Uncoupling protein 2
WBC: White blood cell
WHO-FC: World Health Organization functional class
