# Supplementary material for: Exploratory analysis of the neutrophil to lymphocyte ratio in patients with pulmonary arterial hypertension
Source: BMC Pulm Med. 2017 Apr 26;17:72. doi: 10.1186/s12890-017-0407-5 (PMC5405506; doi:10.1186/s12890-017-0407-5)
Supplement: Supplementary file 1 — Correlation of differential blood count parameters with functional and hemodynamic parameters of patients with PAH. (DOCX 13 kb) [file 12890_2017_407_MOESM1_ESM.docx]

**Supplementary Table 1** Correlation of differential blood count parameters with functional and hemodynamic parameters of patients with pulmonary arterial hypertension (PAH).

| **Parameter** | **6MWD** | **mPAP** | **PVR** | **RAP** | **SvO_2_** | **NT-proBNP** |
| --- | --- | --- | --- | --- | --- | --- |
| Erythrocytes, 10^6^/ml | r=0.216  p=0.056 | r=0.185  p=0.069 | r=0.094  p=0.368 | r=-0.136  p=0.190 | r=0.009  p=0.934 | r=-0.052  p=0.617 |
| Leukocytes, 10^6^/ml | r=-0.139  p=0.222 | r=0.057  p=0.574 | r=-0.019  p=0.858 | r=-0.076  p=0.464 | r=-0.022  p=0.830 | r=0.097  p=0.346 |
| Lymphocytes, 10^6^/ml | r=0.266  p=0.033 | r=-0.088  p=0.446 | r=-0.024  p=0.839 | r=-0.387  p=0.001 | r=0.127  p=0.281 | r=-0.309  p=0.007 |
| Lymphocytes, % | r=0.363  p=0.003 | r=-0.096  p=0.405 | r=0.022  p=0.852 | r=-0.368  p=0.001 | r=0.154  p=0.190 | r=-0.334  p=0.003 |
| Neutrophils, 10^6^/ml | r=-0.256  p=0.041 | r=0.154  p=0.184 | r=0.081  p=0.497 | r=0.136  p=0.252 | r=-0.129  p=0.272 | r=0.230  p=0.048 |
| Neutrophils, % | r=-0.403  p=0.001 | r=0.151  p=0.191 | r=0.096  p=0.418 | r=0.391  p=0.001 | r=-0.155  p=0.188 | r=0.399  p<0.001 |
| Neutrophil / lymphocyte ratio | r=-0.443  p <0.001 | r=0.143  p=0.214 | r=0.116  p=0.327 | r=0.348  p=0.003 | r=-0.209  p=0.073 | r=0.372  p=0.001 |
| Monocytes, 10^6^/ml | r=-0.138  p=0.276 | r=0.060  p=0.605 | r=0.117  p=0.325 | r=0.001  p=0.995 | r=0.027  p=0.818 | r=0.137  p=0.240 |
| Monocytes, % | r=-0.025  p=0.847 | r=-0.076  p=0.513 | r=0.008  p=0.950 | r=0.008  p=0.945 | r=0.162  p=0.171 | r=0.145  p=0.218 |
| Thrombocytes, 10^6^/ml | r=-0.033  p=0.777 | r=-0.231  p=0.023 | r=-0.136  p=0.196 | r=-0.147  p=0.160 | r=0.092  p=0.375 | r=-0.183  p=0.076 |

Pearson correlation was performed to assess parametric data and Spearman’s rank correlation for non-parametric data. Data are presented as correlation coefficients and raw p-values.

6MWD=6 minute walking distance; mPAP=mean pulmonary arterial pressure; NT-proBNP= N-terminal of the prohormone brain natriuretic peptide; PVR=pulmonary vascular resistance; RAP=right atrial pressure; SvO_2_= oxygen saturation of mixed venous blood.
