# Supplementary material for: Exploratory analysis of the neutrophil to lymphocyte ratio in patients with pulmonary arterial hypertension
Source: BMC Pulm Med. 2017 Apr 26;17:72. doi: 10.1186/s12890-017-0407-5 (PMC5405506; doi:10.1186/s12890-017-0407-5)
Supplement: Supplementary file 2 — Association of eosinophils with demographic, functional and hemodynamic as well as with differential blood count parameters in patients with PAH. (DOCX 13 kb) [file 12890_2017_407_MOESM2_ESM.docx]

**Supplementary Table 2** Association of eosinophils with demographic, functional and hemodynamic as well as with differential blood count parameters in patients with pulmonary arterial hypertension (PAH).

| **Parameter** | **Eosinophils** | | **p value** |
| --- | --- | --- | --- |
|  | **≤ 0.1 10^6^ cells/ml** | **> 0.1 10^6^ cells/ml** |  |
| Leukocytes, 10^6^/ml | 7.71 ± 2.02 | 7.82 ± 3.88 | 0.870 |
| Lymphocytes, 10^6^/ml | 1.46 ± 0.59 | 1.62 ± 0.81 | 0.319 |
| Neutrophils, 10^6^/ml | 5.59 ± 2.02 | 5.34 ± 3.79 | 0.730 |
| Age, years | 62 ± 16 | 62 ± 13 | 0.795 |
| Gender, female / male | 30 / 7 | 28 / 12 | 0.260^§^ |
| NYHA I-II / III-IV | 5 / 32 | 10 / 30 | 0.204^§^ |
| 6MWD, m | 317 ± 122 | 390 ± 139 | 0.028 |
| NT-proBNP, ng/l | 3331 ± 4136 | 3222 ± 5307 | 0.221^#^ |
| GFR, ml/min/1.73 m^2^ | 71 ± 47 | 72 ± 53 | 0.596 |
| CRP, > 5 / ≤ 5, mg/dl | 13 / 23 | 27 / 13 | 0.740^§^ |
| mPAP, mm Hg | 49 ± 17 | 38 ± 14 | 0.003 |
| RAP, mmHg | 10 ± 6 | 8 ± 5 | 0.232 |
| PVR, dyn·s·cm^−5^ | 1040 ± 794 | 623 ± 403 | 0.006 |
| SvO_2_, % | 58 ± 13 | 65 ± 11 | 0.009 |
| CI, l/min/m² | 2.2 ± 1.4 | 2.2 ± 0.7 | 0.185 |
| RVSP, mm Hg | 62 ± 23 | 57 ± 23 | 0.403^#^ |
| TAPSE, mm | 17 ± 5 | 19 ± 6 | 0.071 |

Data are presented as mean ± standard deviation or numbers. P-values were calculated by T-test, Mann-Whitney-U^#^ test or Qui-Square test^§^.

6MWD=6 minute walking distance; CI=cardiac index; CRP=C-reactive protein; GFR=glomerular filtration rate; mPAP=mean pulmonary arterial pressure; NT-proBNP= N-terminal of the prohormone brain natriuretic peptide; PVR=pulmonary vascular resistance; RAP=right atrial pressure; RVSP=right ventricular systolic pressure; SvO_2_=oxygen saturation of mixed venous blood; TAPSE=tricuspid annular plane systolic excursion; WHO-FC=World Health Organization functional class.
