# Supplementary material for: Exploratory analysis of the neutrophil to lymphocyte ratio in patients with pulmonary arterial hypertension
Source: BMC Pulm Med. 2017 Apr 26;17:72. doi: 10.1186/s12890-017-0407-5 (PMC5405506; doi:10.1186/s12890-017-0407-5)
Supplement: Supplementary file 3 — Receiver operating characteristics (ROC) across transplantation-free survival: ROC analyses across the range of relative numbers of neutrophils and the neutrophil/lymphocyte ratio in all patients with PAH, in patients with incident PAH and in patients with PAH without cardiovascular risk factors. (PDF 285 kb) [file 12890_2017_407_MOESM3_ESM.pdf]

Supplementary Figure 1

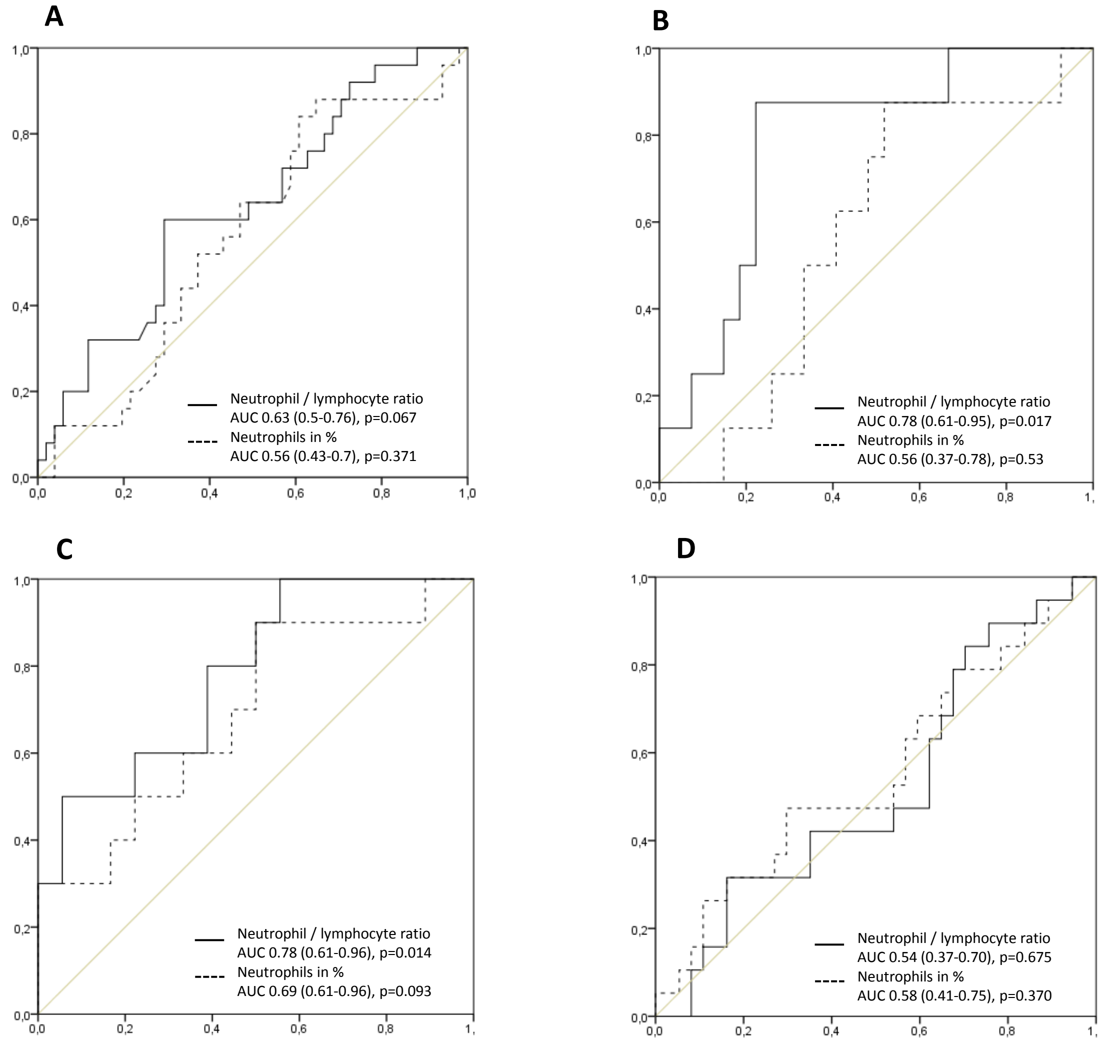

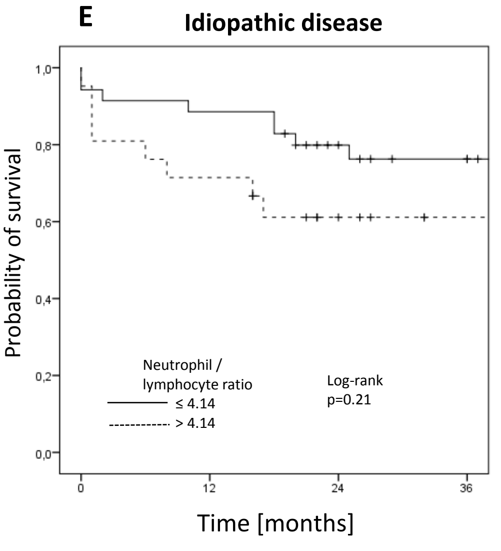

Receiver operating characteristics (ROC) across transplantation-free survival and Kaplan-Meier survival curve in patients with idiopathic disease: ROC analyses across the range of relative numbers of neutrophils and the neutrophil / lymphocyte ratio in all patients with PAH (**A**), in patients with incident PAH (**B**), in patients with PAH without cardiovascular risk factors (**C**) and in patients with idiopathic disease (**D**). Transplantation-free survival in patients with idipathic disease (**E**).
