# Supplementary material for: Exploratory analysis of the neutrophil to lymphocyte ratio in patients with pulmonary arterial hypertension
Source: BMC Pulm Med. 2017 Apr 26;17:72. doi: 10.1186/s12890-017-0407-5 (PMC5405506; doi:10.1186/s12890-017-0407-5)
Supplement: Supplementary file 4 — Association of the neutrophil/lymphocyte ratio with demographic, functional and hemodynamic parameters, as well as co-morbidities. (DOCX 16 kb) [file 12890_2017_407_MOESM4_ESM.docx]

**Supplementary Table 3** Association of the neutrophil / lymphocyte ratio with demographic, functional and hemodynamic parameters, as well as co-morbidities.

| **Parameter** | **Neutrophil / lymphocyte ratio** | | **p-value** |
| --- | --- | --- | --- |
|  | **≤ 4.14** | **> 4.14** |  |
| Age, years | 58 ± 15 | 68 ± 12 | 0.004 |
| Gender, female / male | 38 / 9 | 20 / 10 | 0.159^§^ |
| NYHA I -II / III-IV | 12 / 35 | 3 / 37 | 0.093^§^ |
| 6MWD, m | 398 ± 126 | 272 ± 113 | <0.001 |
| NT-proBNP, ng/l | 2134 ± 3490 | 4983 ± 5834 | 0.001^#^ |
| GFR, ml/min/1.73 m^2^ | 61 ± 35 | 88 ± 64 | 0.020 |
| CRP, > 5 / ≤ 5, mg/dl | 37 / 10 | 13 / 16 | 0.002^§^ |
| mPAP, mm Hg | 41 ± 18 | 46 ± 14 | 0.192 |
| RAP, mmHg | 7 ± 4 | 12 ± 6 | <0.001 |
| PVR, dyn·s·cm^−5^ | 829 ± 741 | 811 ± 463 | 0.908 |
| SvO_2_, % | 63 ± 12 | 59 ± 13 | 0.248 |
| CI, l/min/m² | 2.1 ± 0.7 | 2.5 ± 1.6 | 0.152 |
| RVSP, mm Hg | 60 ± 25 | 59 ± 19 | 0.306 |
| TAPSE, mm | 19 ± 6 | 16 ± 6 | 0.066 |
| Arterial hypertension | 18 (38%) | 14 (47%) | 0.467^§^ |
| Diabetes mellitus | 2 (4%) | 8 (27%) | 0.004^§^ |
| Coronary heart disease | 6 (13%) | 6 (20%) | 0.393^§^ |
| Atrial fibrillation | 8 (17%) | 7 (23%) | 0.495^§^ |
| BMI ≥ 30 kg/m² | 9 (19%) | 8 (27%) | 0.438^§a^ |
| CVRF ≥ 3^&^ | 2 (4%) | 5 (16%) | 0.065^§^ |
| Chronic obstructive pulmonary disease | 13 (28%) | 3 (10%) | 0.063^§^ |

Data are presented as mean ± standard deviation or numbers. P-values were calculated by T-test, Mann-Whitney-U^#^ test or Qui-Square test^§^. ^&^CVRF including arterial hypertension, diabetes mellitus, coronary heart disease, atrial fibrillation and body mass index.

6MWD=6 minute walking distance; BMI=body mass index; CI=cardiac index; CRP=C-reactive protein; CVRF=cardiovascular risk factors; GFR=glomerular filtration rate; mPAP=mean pulmonary arterial pressure; NT-proBNP= N-terminal of the prohormone brain natriuretic peptide; PVR=pulmonary vascular resistance; RAP=right atrial pressure; RVSP=right ventricular systolic pressure; SvO_2_=oxygen saturation of mixed venous blood; TAPSE=tricuspid annular plane systolic excursion; WHO-FC=World Health Organization functional class.
